# PROTECT: Prospective Phase-II-Trial Evaluating Adaptive Proton Therapy for Cervical Cancer to Reduce the Impact on Morbidity and the Immune System

**DOI:** 10.3390/cancers13205179

**Published:** 2021-10-15

**Authors:** Anouk Corbeau, Remi A. Nout, Jan Willem M. Mens, Nanda Horeweg, Jérémy Godart, Ellen M. Kerkhof, Sander C. Kuipers, Mariette I. E. van Poelgeest, Judith R. Kroep, Ingrid A. Boere, Helena C. van Doorn, Mischa S. Hoogeman, Uulke A. van der Heide, Hein Putter, Marij J. P. Welters, Sjoerd H. van der Burg, Carien L. Creutzberg, Stephanie M. de Boer

**Affiliations:** 1 Department of Radiation Oncology, Leiden University Medical Center, Albinusdreef 2, 2333 ZA Leiden, The Netherlands; n.horeweg@lumc.nl (N.H.); e.m.kerkhof@lumc.nl (E.M.K.); u.a.van_der_heide@lumc.nl (U.A.v.d.H.); c.l.creutzberg@lumc.nl (C.L.C.); s.m.de_boer.onco@lumc.nl (S.M.d.B.); 2 Department of Radiotherapy, Erasmus MC Cancer Institute, Dr. Molewaterplein 40, 3015 GD Rotterdam, The Netherlands; r.nout@erasmusmc.nl (R.A.N.); j.w.m.mens@erasmusmc.nl (J.W.M.M.); j.schiphof-godart@erasmusmc.nl (J.G.); s.kuipers@erasmusmc.nl (S.C.K.); m.hoogeman@erasmusmc.nl (M.S.H.); 3 HollandPTC, Huismansingel 4, 2629 JH Delft, The Netherlands; 4 Department of Gynecology and Obstetrics, Leiden University Medical Center, Albinusdreef 2, 2333 ZA Leiden, The Netherlands; m.i.e.van_poelgeest@lumc.nl; 5 Department of Medical Oncology, Leiden University Medical Center, Albinusdreef 2, 2333 ZA Leiden, The Netherlands; j.r.kroep@lumc.nl (J.R.K.); m.j.p.schoenmaekers-welters@lumc.nl (M.J.P.W.); s.h.van_der_burg@lumc.nl (S.H.v.d.B.); 6 Department of Medical Oncology, Erasmus MC Cancer Institute, Dr. Molewaterplein 40, 3015 GD Rotterdam, The Netherlands; i.boere@erasmusmc.nl; 7 Department of Gynecological Oncology, Erasmus MC Cancer Institute, Dr. Molewaterplein 40, 3015 GD Rotterdam, The Netherlands; h.vandoorn@erasmusmc.nl; 8 Department of Medical Statistics, Leiden University Medical Center, Albinusdreef 2, 2333 ZA Leiden, The Netherlands; h.putter@lumc.nl; 9 Oncode Institute, Jaarbeursplein 6, 3521 AL Utrecht, The Netherlands

**Keywords:** cervical cancer, proton therapy, chemoradiotherapy, dose reduction, bone marrow, bowel, toxicity, quality of life

## Abstract

**Simple Summary:**

Chemoradiation with photon radiotherapy is very effective as a locally advanced cervical cancer (LACC) treatment. However, the majority of women with LACC experience treatment-related toxicity involving the gastrointestinal and urogenital tracts and the immune system. Compared to that of photon therapy, proton therapy substantially reduces undesired dose to the organs around the tumor, leading to a decrease in radiotherapy-related side-effects. At present, few studies on proton therapy in patients with LACC will be conducted. The PROTECT trial aims to evaluate the differences in side effects between photon therapy and proton therapy, both combined with chemotherapy, for LACC. Fifteen patients will be enrolled per treatment group. Information will be collected on the differences in dose to the organs around the tumor, treatment-related side effects, and the impact on the immune system. This information will be used to assess the potential of proton therapy as an innovative treatment for LACC.

**Abstract:**

External beam radiation therapy (EBRT) with concurrent chemotherapy followed by brachytherapy is a very effective treatment for locally advanced cervical cancer (LACC). However, treatment-related toxicity is common and reduces the patient’s quality of life (QoL) and ability to complete treatment or undergo adjuvant therapies. Intensity modulated proton therapy (IMPT) enables a significant dose reduction in organs at risk (OAR), when compared to that of standard intensity-modulated radiation therapy (IMRT) or volumetric-modulated arc therapy (VMAT). However, clinical studies evaluating whether IMPT consequently reduces side effects for LACC are lacking. The PROTECT trial is a nonrandomized prospective multicenter phase-II-trial comparing clinical outcomes after IMPT or IMRT/VMAT in LACC. Thirty women aged >18 years with a histological diagnosis of LACC will be included in either the IMPT or IMRT/VMAT group. Treatment includes EBRT (45 Gy in 25 fractions of 1.8 Gy), concurrent five weekly cisplatin (40 mg/m^2^), and 3D image (MRI)-guided adaptive brachytherapy. The primary endpoint is pelvic bones D_mean_ and mean bowel V_15Gy_. Secondary endpoints include dosimetric parameters, oncological outcomes, health-related QoL, immune response, safety, and tolerability. This study provides the first data on the potential of IMPT to reduce OAR dose in clinical practice and improve toxicity and QoL for patients with LACC.

## 1. Introduction

Cervical cancer is the most frequent gynecological cancer worldwide, with an estimated 604,000 newly diagnosed patients and 342,000 cancer-related deaths in 2020 [1]. In the Netherlands, the incidence has slightly increased from about 700–750 to more than 900 over the past years [2]. Approximately 30% of these women present with locally advanced disease (LACC), wherein the tumor invades surrounding tissues and/or spreads to regional lymph nodes [3]. The current standard treatment for LACC combines external beam radiation therapy (EBRT) with concurrent platinum-based chemotherapy followed by MR-guided brachytherapy [4]. This combination of treatment modalities was proven highly effective for local and pelvic tumor control and improved survival in patients with LACC significantly over the last three decades [3,5,6,7]. In a previous analysis, we showed a 5-year local control, pelvic control, and cancer specific survival of respectively 90.4%, 82.4%, and 72.7% for women with LACC, which is very comparable to the outcomes of the prospective EMBRACE-I trial [6,8]. Since the majority of patients are young and currently have the prospect of long-term survival after treatment, late treatment-related morbidity can have a profound impact on their quality of life (QoL) [9,10,11]. In our cohort, 67.7% of the women suffered from mild to moderate toxicity (grade 1–2), and severe morbidity (grade 3) occurred in 8–10% of the patients [6,7,8,12]. A total of 14.6% of the patients in the EMBRACE-I trial had grade 3–5 adverse events [8]. The most commonly reported severe late toxicities concern the gastro-intestinal and urogenital tracts and insufficiency fractures of bones in the irradiated area [6,8,10,13,14]. In our cohort, cumulative incidences of severe late bladder, vaginal, rectal, bowel, and bone toxicity were 0.8%, 1.4%, 3.3%, 3.6%, and 2.8% respectively at 5 years of follow-up [6]. Actuarial cumulative 5-year incidence of grade 3–5 morbidity in the EMBRACE-I trial was 6.8% for genitourinary events, 8.5% for gastrointestinal events, 5.7% for vaginal events, and 3.2% for fistulae [8].

Various parts of the gastrointestinal tract are being exposed during pelvic EBRT, consequently causing gastrointestinal morbidities. Studies found the V_15Gy_ of the small bowel to be correlated with acute bowel toxicity [15,16]. Besides, conventional EBRT combined with chemotherapy has a clear suppressive impact on the immune system [17]. Conventional EBRT with cisplatin significantly decreased the absolute numbers of circulating leukocytes and lymphocytes, which could last for more than nine weeks. Additionally, an increase in immune suppressive myeloid-derived suppressor cells (MDSCs) and a decreased T-cell function were observed [17]. Immunosuppression may negatively impact the efficacy, feasibility, and safety of conventional chemotherapy and (future) adjuvant immune therapies. Reduction of the immunosuppressive impact of chemoradiation might improve the tolerance for treatment of LACC and facilitate an increase in treatment intensity for high-risk patients [18]. Studies investigating dose-effect relationships found the V_10Gy_, V_20Gy_, and V_40Gy_ of the whole pelvic bones to be most strongly associated with hematologic toxicity for patients with LACC undergoing cisplatin-based chemoradiation [19,20,21,22,23].

**Figure 1:**
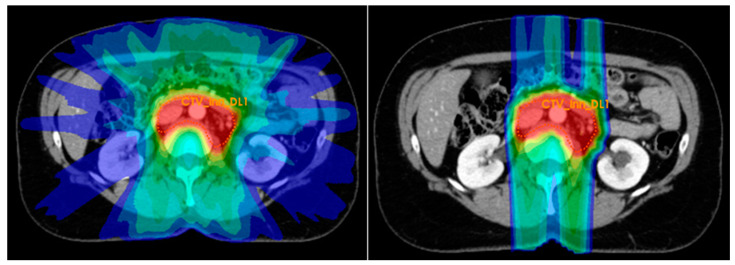
A typical dose distribution for the para-aortic region for IMRT (**left**) and IMPT (**right**) [24].

With proton therapy (PT), highly localized dose deposition is possible by making use of protons’ finite range. This enables a significant reduction in the dose to organs at risk (OAR) without compromising target volume coverage. A treatment planning study from our group demonstrated that intensity modulated proton therapy (IMPT) significantly reduced both the D_mean_ of the pelvic bones with 29% and the V_15Gy_ of the bowel bag with 28%, when compared to that of intensity modulated radiation therapy (IMRT) [24]. In Figure 1 typical dose distributions for para-aortic target volumes is shown, with steep dose fall-off and excellent sparing of the bowel bag and kidneys with IMPT. Gort et al. compared robustly optimized pencil beam scanning (PBS) PT with volumetric modulated arc therapy (VMAT) and showed similar target coverage robustness, while PBS PT plans offered similar or significantly better OAR sparing [25]. It should be investigated whether these lower OAR doses result in an actual toxicity reduction.

Early studies demonstrated the feasibility of treating gynecological cancer patients with proton therapy [26,27]. Even though these results are encouraging, clinical data on our population of interest are sparse since these studies included only small groups of patients with diverse gynecological cancers and concurrent treatments. Clinical studies evaluating IMPT for LACC are warranted to assess the potential of the technique for treatment of LACC [28].

The aim of this prospective study is to investigate in clinical practice the differences between IMPT and IMRT or VMAT photon therapy in the effects on dose-volume parameters and treatment-related morbidity for women with LACC undergoing chemoradiation. Since the PROTECT trial allows for the development and implementation of IMPT for LACC, the safety, tolerability, and outcomes of the technique will be assessed as well. Moreover, the bone marrow sparing capability and effects on the local and systemic immune response of the radiotherapeutic techniques will be evaluated. 

## 2. Materials and Methods

### 2.1. Study Design

**Figure 2:**
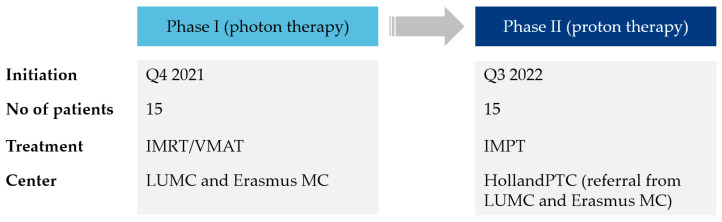
Schematic scheme of PROTECT’s study design. IMRT, intensity-modulated radiation therapy; VMAT, volumetric-modulated arc therapy; IMPT, intensity modulated proton therapy.

The study is designed as a prospective, multicenter, nonrandomized phase-II trial to compare IMPT with IMRT/VMAT with photons in patients with LACC treated with pelvic +/− para-aortic radiotherapy combined with concurrent chemotherapy with curative intent. During the first phase of the trial, 15 patients will be enrolled in the IMRT/VMAT treatment group. In the second phase of the trial, 15 patients will be enrolled in the IMPT group. Figure 2 provides an overview of the study design.

### 2.2. Patient Selection

Patients aged 18 years or older with a histological diagnosis of LACC with pelvic and/or para-aortic lymph node involvement (according to the EMBRACE-II guidelines [29]) and an indication for curative treatment with primary chemoradiation (with cisplatin) followed by 3D image (MRI)-guided adaptive brachytherapy are eligible. All of the inclusion criteria in Table 1 have to be met. A potential subject who meets any of the exclusion criteria in Table 1 will not be enrolled in this study.

### 2.3. Study Objectives

The primary objective of this trial is to investigate whether in clinical practice IMPT can significantly reduce the D_mean_ to the pelvic bones and the mean V_15Gy_ to the bowel compared to that of IMRT/VMAT with photons. Secondary objectives are to compare IMPT with standard of care IMRT/VMAT with respect to dosimetric parameters, clinical outcomes, health-related quality of life, safety, and tolerability. Furthermore, the effect of IMPT and IMRT/VMAT on the local and systemic immune system are evaluated, as measured by the bone marrow activity on MRI scans with Dixon technique and the number and function of circulating leukocytes (myeloid cells and lymphocytes).

### 2.4. Trial Organization and Coordination

The PROTECT study is a collaborative project between Erasmus Medical Center (Erasmus MC), Leiden University Medical Center (LUMC), and Holland Proton Therapy Center (HPTC) in the Netherlands. Patients will receive IMRT/VMAT at Erasmus MC or LUMC. The treatment with IMPT will take place at HPTC. The clinical development and implementation of IMPT for treatment of LACC is being performed by Erasmus MC, since the PROTECT study facilitates the first chemoradiation treatment with IMPT for LACC in the Netherlands. The clinical trial wherein IMPT is implemented and compared to IMRT/VMAT is led by LUMC.

### 2.5. Ethics, Informed Consent, and Safety

The study protocol is under consideration for approval by the Leiden-The Hague-Delft Medical Ethics Committee (LDD-METC). This study complies with the standards of Good Clinical Practice, the Helsinki Declaration, the Dutch law, and Medical Research Involving Human Subjects Act (WMO). The trial is registered in the Netherlands Trial Register with the registration number NL 9567.

### 2.6. Sample Size Calculation and Statistical Analysis

**Figure 3:**
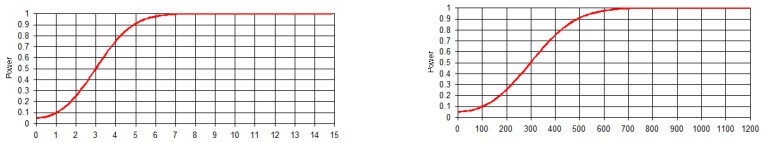
Sample size calculation to determine power to detect significant differences in pelvic bones D_mean_ (Gy (**left**) and bowel mean V_15Gy_ (cc) (**right**) when comparing IMPT and IMRT/VMAT.

Sample size calculations were performed to determine the power of this study to be able to detect differences between IMPT and IMRT/VMAT. Compared with that of IMRT/VMAT, IMPT is expected to cause bigger reductions in dose to pelvic bones and bowel when the para-aortic region is included in the target volume. To detect significant differences between the two treatments and to keep the groups comparable, the minimum and maximum number of patients receiving para-aortic radiotherapy is respectively 7 and 10 in each treatment group. When including 7 patients receiving para-aortic radiotherapy and 8 patients receiving iliac radiotherapy in each treatment group, a difference of 12.82 Gy and 632.47 cc in respectively the pelvic bones D_mean_ and bowel mean V_15Gy_ is expected [24]. Figure 3 visualizes that with the inclusion of 15 evaluable patients in each treatment group (two-sided α = 0.05), there is 80.8% power to detect a true difference in the pelvic bones D_mean_ of 4.21 Gy and 79.2 % power to detect a true difference in bowel mean V_15Gy_ of 400 cc.

Differences in dosimetric parameters will be analyzed using a linear regression with a correction for the target volume (pelvic common iliac lymph nodes with or without para-aortic lymph nodes). With respect to differences in clinical outcomes between the two groups, the proportion of patients with a complete response at three months after treatment will be compared with a chi-square or fisher exact test. Kaplan–Meier log-rank test will be used for the overall survival, local, pelvic, and distant recurrence-free survival. For the evaluation of patient reported symptoms and QoL, the European Organization for Research and Treatment of Cancer (EORTC)-core (C-30) questionnaire, the CX24 module for cervical cancer, and six additional questions from EN24 module will be used. Outcomes will be described and compared between the two groups. Toxicity will be graded according to the NCI-CTCAE version 5.0 [30]. Lastly, the differences in immune parameters over time will be analyzed with a mixed model analysis of variance including time, chemotherapy and chemotherapy by time as fixed effects and the subjects as random effects. SPSS and R will be used for statistical analysis. A *p*-value < 0.05 will be considered statistically significant. This comparative study is considered successful when both the pelvic bones D_mean_ and mean bowel V_15Gy_ are significantly lower with IMPT than with IMRT/VMAT.

### 2.7. Investigation Schedule

#### 2.7.1. Preregistration Evaluation Procedures

The appropriate treatment for each patient is based on interdisciplinary assessment following approved diagnostic procedures and treatment guidelines [4,29]. Staging is based on physical examination, a pelvic MRI scan, a chest CT or radiograph, and an optional total body PET-CT scan. The baseline toxicity and QoL will be evaluated. 

#### 2.7.2. External Beam Radiation Therapy Planning

For external beam radiation therapy, the use of a ‘library of plans’ technique with daily selection of the most appropriate treatment plan using cone beam CT (CBCT) is standard of care in the participating centers [31]. CT planning scans in treatment position with full and empty bladder will be obtained and merged to create an internal target volume (ITV) accounting for movement of the uterus and cervical vault region by variations in bladder and rectal filling. OAR include the rectum, sigmoid, bowel bag, bladder, femoral heads, kidneys, spinal cord, and the whole pelvic bone contour. Dose coverage, planning aims, and hard dose constraints for the OAR will be according to the EMBRACE-II protocol [29]. Since the specific goal in this project is to aim for simultaneous bone marrow and bowel sparing, treatment planning of both IMPT and IMRT/VMAT will be optimized for bone marrow and bowel sparing.

#### 2.7.3. External Beam Radiation Therapy Delivery

EBRT is given to a total dose of 45 Gy in 25 daily fractions of 1.8 Gy in 5 weeks. Involved nodes are boosted using a simultaneous integrated boost (SIB) to reach a total EBRT plus brachytherapy dose of 60 Gy EQD2 to provide high nodal control [32]. CBCT scans or, when available, in-room CT scans are used for daily evaluation of patient positioning and the selection of the most appropriate treatment plan. 

#### 2.7.4. Brachytherapy

Brachytherapy is performed using a high-dose rate (HDR) after loading system to deliver a boost to any residual tumor and the cervix. Brachytherapy dose is (21-) 28 Gy in fractions of 7 Gy specified at 100% isodose around the high-risk CTV, according to the EMBRACE-II prescription protocol. The aim is to reach an equivalent dose in 2 Gy fractions including EBRT (EQD2_D90) of the high-risk CTV between 90–95 Gy, using MRI-guided adaptive brachytherapy [29]. The maximum overall treatment time including EBRT and brachytherapy is 50 days.

#### 2.7.5. Chemotherapy

The standard chemotherapy regimen is weekly cisplatin (40 mg/m^2^) for 5 weeks. Reduced chemotherapy dose to 75% per cycle is permitted when the full dose cannot be given.

#### 2.7.6. Monitoring during Treatment and Follow-Up

**Figure 4:**
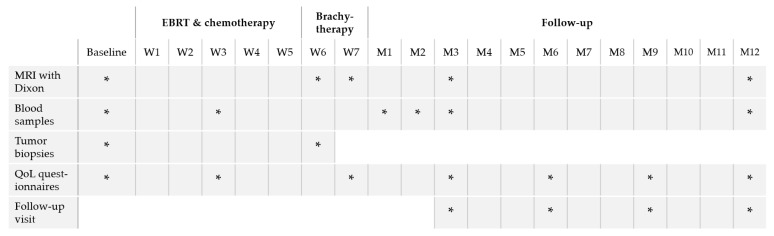
Overview of data collection points per patient enrolled in the PROTECT trial. *, data collection point; EBRT, external beam radiation therapy; W, week; M, month; MRI, magnetic resonance imaging; QoL, quality of life.

In Figure 4, an overview of the timepoints for data collection per patient is shown.

Patients will have an MRI scan with Dixon technique for evaluation of bone marrow activity at baseline, for all brachytherapy purposes, and at 12 weeks and 12 months after treatment. Blood samples will be collected at baseline, week 3 of treatment, and 4, 8, and 12 weeks and 12 months after treatment for immune-monitoring using the same methods described previously by our group [17]. Tumor biopsies will be collected at baseline and at the first brachytherapy session for evaluation of the impact of treatment on the local immune response. QoL questionnaires will be completed at baseline, week 3 of EBRT, at completion of brachytherapy and at 3, 6, 9, and 12 months after treatment.

Four weeks after treatment, the first clinical response assessment will be done. If applicable, hormonal replacement therapy, vaginal dilators, and vaginal estrogens will be prescribed as per standard practice. Tumor and nodal remission will be assessed as complete, uncertain complete, partial, stable, or progressive disease at three months after treatment by gynecological examination and a pelvic MRI scan and/or thoraco-abdominal-pelvic CT scan. When remission is uncertain, imaging will be repeated after 2–3 months and, if applicable, finally histological confirmation will be obtained in case of persistent or recurrent disease.

Thereafter, patients will be followed up at 3-month intervals by the radiation oncologist and gynecologic oncologist during the first few years. At each follow-up visit, tumor recurrence and treatment-related morbidity will be assessed by targeted patient history and physical and pelvic examination. Additional imaging will only be performed in case of symptoms or suspicion of recurrence.

### 2.8. Duration of the Study

The accrual of the patients for IMRT/VMAT is expected to be completed within one year. The subsequent accrual of the patients for the proton therapy group is expected to be completed within 1.5 years. The study ends one year after the last patient completes treatment.

## 3. Discussion

The PROTECT trial is the first prospective clinical trial to compare IMPT with IMRT/VMAT for women with LACC treated with primary chemoradiation. The study is expected to yield a wealth of information on the differences in dose-volume parameters, physician and patient-reported morbidity, and the immune system. Compared to that of IMRT/VMAT, proton therapy is expected to substantially reduce the unwanted exposure of OAR to radiation dose for women with LACC, while maintaining the high level of disease control. Resulting reductions in morbidity have the potential to importantly improve QoL and functioning of cancer survivors. As women with LACC are most often diagnosed in the early decades of their lives, these improvements are of major societal relevance. Moreover, this clinical study creates a unique opportunity to study the effects of both types of radiation therapy on the local and systemic immune response. The bone marrow sparing effect of proton therapy may reduce the considerable immune suppressive impact of the current IMRT/VMAT photon techniques, which may in turn increase the likelihood of safe and effective delivery of additional systemic (immune) therapies. This is especially relevant in patients at high risk of distant metastasis. With the PROTECT trial, the potential of proton therapy for LACC to positively influence the chemoradiation treatment tolerance will be assessed. In the future, IMPT might facilitate treatment intensification, i.e., by the addition of adjuvant immune therapies to the treatment of high-risk patients.

The use of proton beam therapy is increasing. Since proton therapy is more expensive than photon therapy, it is important to identify the (sub)population of each cancer type for whom proton therapy is most cost effective [33]. Several tumor types, including intra-ocular, base-of-skull, low-grade brain tumors, and pediatric cancers, have a standard indication for IMPT in the Netherlands. For other tumor types, including head-and-neck, breast, esophageal and lung cancers, and lymphomas, normal tissue complication probability (NTCP) models are used to select patients who are expected to benefit significantly from proton therapy [34]. Currently, patients with pelvic and gynecological cancers have no indication for proton therapy in the Netherlands. The PROTECT trial will allow for the development and implementation of proton therapy for LACC. Planning studies demonstrated that IMPT could significantly reduce the dose to OAR when compared to that of IMRT/VMAT. This phase-II trial is the next step to evaluate whether these dose differences are feasible in clinical practice. Since the patients included in this study will be the first to undergo IMPT for pelvic cancer in the Netherlands, the safety, tolerability, and short- and long-term outcomes of the technique are also evaluated. Our results will provide essential data to develop indications and guidelines for proton therapy for LACC.

Existing data on outcomes and toxicities of proton therapy in LACC are limited. The earliest studies investigated proton therapy as an alternative to brachytherapy or used older techniques for proton therapy delivery [35,36,37]. Two other studies demonstrated the feasibility of proton therapy for gynecological cancer treatment. A small, prospective single-arm study by Lin et al. evaluated 11 patients with gynecological cancer treated posthysterectomy and showed that pencil beam scanning (PBS) PT was able to reduce the dose to the OAR substantially compared to IMRT plans [26]. Xu et al. demonstrated that PBS PT resulted in significantly lower volumes of exposed bone marrow, small bowel, and bladder when dosimetrically comparing PBS PT with IMRT for endometrial cancer [27]. An ongoing trial is the APROVE study, which is a prospective single-center one-arm study wherein 25 gynecological cancer patients with an indication for postoperative pelvic radiotherapy will be treated with PBS PT [38]. The aim of this study is to explore the advantages of proton therapy in radiotherapy for gynecological cancers. In contrast to the APROVE study, our trial uses a control group to compare IMPT with IMRT/VMAT for women with LACC treated with primary chemoradiation. When the PROTECT trial provides the first data on the potential of IMPT to reduce OAR dose and improve toxicity and QoL for patients with LACC, additional prospective studies with bigger cohorts and longer follow-up time will be needed to verify and substantiate these findings.

## 4. Conclusions

The prospective PROTECT trial investigates the improvement in dose to OAR, especially bowel and bone marrow, and QoL with IMPT compared to that of IMRT/VMAT in patients with LACC undergoing chemoradiation. The safety, tolerability, and short- and long-term outcomes of IMPT are also assessed. Moreover, the bone marrow sparing capability and effects on the local and systemic immune system of these two radiotherapeutic techniques will be evaluated. In this way, the improvement of outcomes with proton therapy for primary treatment of women with LACC can be substantiated.

## Figures and Tables

**Table 1:** Inclusion and exclusion criteria for participation in the PROTECT trial.

**Inclusion Criteria**
Histological diagnosis of LACC; Curative treatment with chemoradiation (with cisplatin or alternatively carboplatin); Aged 18 years or older; WHO performance status 0–1; Indication to include the common iliac region +/− the para-aortic region into the elective clinical target volume of the EBRT; No distant metastasis beyond the para-aortic lymph node chain; Adequate systemic organ function: ∘ Creatinine clearance (>50 cc/min), ∘ Adequate bone marrow function: white blood cells ≥ 3.0 × 10^9^/L, neutrophils ≥ 1.5 × 10^9^/L, platelets ≥ 100 × 10^9^/L.
**Exclusion Criteria**
Another primary malignancy active or present within the last 5 years; Other severe diseases such as recent myocardial infarction; Previous pelvic or abdominal radiotherapy; Previous major abdominal/pelvic surgery for LACC; Neo-adjuvant chemotherapy treatment; (History of) active primary immunodeficiency or autoimmune disorder; Use of immunosuppressive drugs at baseline.

LACC, locally advanced cervical cancer; WHO, World Health Organization; EBRT, external beam radiation therapy.
